# Competitive allele-specific TaqMan PCR (Cast-PCR) is a sensitive, specific and fast method for BRAF V600 mutation detection in Melanoma patients

**DOI:** 10.1038/srep18592

**Published:** 2015-12-22

**Authors:** Raffaela Barbano, Barbara Pasculli, Michelina Coco, Andrea Fontana, Massimiliano Copetti, Michelina Rendina, Vanna Maria Valori, Paolo Graziano, Evaristo Maiello, Vito Michele Fazio, Paola Parrella

**Affiliations:** 1 Laboratory of Oncology, IRCCS Casa Sollievo della Sofferenza, San Giovanni Rotundo (FG) 71013, Italy; 2 Biostatic Unit, IRCCS Casa Sollievo della Sofferenza, San Giovanni Rotundo (FG) 71013, Italy; 3 Department of Oncology, IRCCS Casa Sollievo della Sofferenza, San Giovanni Rotundo (FG) 71013, Italy; 4 Department of Pathology, IRCCS Casa Sollievo della Sofferenza, San Giovanni Rotondo (FG) 71013, Italy; 5 CIR Laboratory for Molecular Medicine and Biotechnology, University Campus Biomedico, Rome 00128, Italy

## Abstract

*BRAF* codon 600 mutation testing of melanoma patients is mandatory for the choice of the most appropriate therapy in the clinical setting. Competitive allele specific TaqMan PCR (Cast-PCR) technology allows not only the selective amplification of minor alleles, but it also blocks the amplification of non-mutant allele. We genotyped codon 600 of the *BRAF* gene in 54 patients’ samples by Cast-PCR and bidirectional direct sequence analysis. All the mutations detected by sequencing were also identified by Cast-PCR. In addition, Cast-PCR assay detected four samples carrying mutations and was able to clearly identify two mutations of uncertain interpretation by Sanger sequencing. The limit of detection of Cast-PCR was evaluated by constructing dilution curves of BRAF^V600E^ and BRAF^V600K^ mutated clinical samples mixed with a not-mutated specimens. Both mutations could be detected until a 1:100 mutated/not mutated ratio. Cloning and sequencing of the clones was used to confirm mutations on representative discrepant cases. Cast PCR performances were not affected by intratumour heterogeneity, and less affected by melanin content. Our results indicate that Cast-PCR is a reliable diagnostic tool for the identification of melanoma patients as eligible to be treated with TKIs and might be implemented in the clinical setting as elective screening method.

Melanomas are malignant tumours arising from the pigment producing cells melanocytes which are derived from the neural crest and are widely distributed throughout all cutaneous, ocular and most of the mucosal surfaces^1^,^2^,^3^,^4^. It has long been known that activation of the RAS–RAF–MEK–ERK–MAPK (mitogen activated protein kinase, MAPK) pathway plays an important role in melanoma development^5^. In particular, somatic mutations of the *BRAF* gene encoding for a serine/threonine kinase that transduces regulatory signals from RAS through MEK to MAPK are detected in 40–60% of melanoma cases^6^,^7^,^8^,^9^,^10^. The most common mutations of the *BRAF* gene in melanoma occur within exon 15 codon 600 are the *BRAF*^V600E^(c.1799 T > A, p.Val600Glu), as detected in approximately 80% of mutated cases, and the *BRAF*^V600K^ (c.1798_1799delinsAA, p. Val600Lys), detected in 5–30% of mutated cases^10^,^11^.

Although cutaneous melanoma accounts for only 4% of all dermatologic cancers, it is responsible for 80% of the deaths^2^. Patients with localized melanoma have a 5-year survival rate of about 98%, but this rate radically declines in presence of regional or distant metastases to reach 63% and 16%, respectively^3^. The prognosis of metastatic melanoma has recently changed substantially thanks to the approval of the kinase inhibitors Vemurafenib^8^,^9^,^10^,^11^,^12^ and Dabrafenib^13^,^14^,^15^, and the immune checkpoint inhibitor Ipilimumab^16^,^17^.

Vemurafenib^8^,^9^,^10^,^11^,^12^ and Dabrafenib^13^,^14^,^15^,^16^,^17^ are selective inhibitors of *BRAF* codon 600 mutations, and are approved by the US Food and Drug Administration (FDA) and the European Medicines Agency (EMA) for the treatment of unresectable or metastatic melanoma. The demonstration of clinical activity for BRAF inhibitors has created the need for accurate, robust, rapid and cost-effective *BRAF* mutation screening assays.

Several techniques are currently used to detect *BRAF* mutations, and they can be divided into screening methods, able to identify mutations in the entire amplified region, and targeted methods, which specifically amplify mutated amplicons. Among the screening methods, bidirectional sequencing (Sanger sequencing) and pyrosequencing are the most widely used in the clinical setting, whereas allele-specific PCR (TaqMan) is the most common targeted method. In addition, MALDI-TOF MS (Matrix-assisted laser desorption/ionization time of flight mass spectrometry) and Next Generation Sequencing hold promises for the identification of multiple somatic mutations. All these methods have been validated in terms of analytical sensitivity, specificity, limit of detection, costs, and response delay. In particular, as regards the screening methods, the limit of detection is 20% for Sanger sequencing and 5–10% for pyrosequencing, whereas the limit of detection of Real Time PCR- based targeted methods is 1% or less^18^,^19^,^20^,^21^. However, the actual diagnostic performance of each technique should be evaluated in multiple independent cohorts as being of crucial importance before their implementation into the clinical setting. Indeed, *BRAF* mutation analysis in melanoma has several limitations as compared with other tumour types because of the melanin content which interferes with PCR reaction, and the intratumour heterogeneity as reported by several studies^22^,^23^,^24^,^25^. Thus, there is the need to develop diagnostic protocols able to address these issues for reducing the risks of both false positive and false negative results.

Competitive allele-specific hydrolysis probes (TaqMan) PCR is a modification of the Taqman based allele specific PCR which works by combining a mutant allele-specific primer (ASP) with a wild type allele-specific blocker (ASB) in the same PCR reaction. The use of a molecular blocker completely suppresses the amplification of the wild type allele in order to not interfere with the amplification of the mutant allele and improve the specificity as well as the sensitivity of the assay. In a double blinded study comparing direct sequencing, single-strand conformation analysis (SSCA), high resolution melting analysis (HRM) and Cast-PCR^27^, the latter was shown to be a sensitive and rapid method for *BRAF* mutation detection.

Here, we report the analysis by Cast-PCR and Sanger sequencing of 54 clinical samples as part of the routine clinical screening for *BRAF* codon 600 mutations. To mimic very diagnostic conditions, the sensitivity of both Sanger sequencing and Cast-PCR was evaluated by using patients’ DNA samples harbouring either BRAF^V600E^ or BRAF^V600K^ mutations respectively, diluted with a not mutated DNA sample at different ratios. The specificity of the assay was determined by cloning and sequencing of the clones on representative discrepant cases. To the best of our knowledge, no data about the effects of melanin content on the analytical procedures in melanoma samples are currently available. Thus, we additionally evaluated the diagnostic performance of Cast-PCR and Sanger sequencing in samples before and after purification with glass milk^28^.

## Results

### Comparison between Cast-PCR and Sanger sequencing for the detection of BRAF^V600E^ and BRAF^V600K^ in clinical samples

κ−statistic showed a significant agreement between the two techniques (κ = 0.85, CI: 0.710–0.990; *p* < 0.001). As shown in Table 1, all cases identified as mutated by Sanger analysis were confirmed by Cast-PCR. Moreover, the latter definitely identified as mutated two cases showing low height peaks by Sanger sequencing (case 6 and case 22), and demonstrated the presence of mutation in one case (case 25) in which direct sequencing failed. Importantly, Cast-PCR identified additional 4 mutated samples (case 1, case 5, case 7 and case 11) as compared with Sanger sequencing (Table 2). The calculated positive agreement considering Cast-PCR as the reference method was 85.7% (CI95%: 67.3–96.7%) and the negative agreement was 100% (CI95%: 86.3–100%) (Table 3).

### Cast-PCR is a high sensitive method for the detection of BRAF Val600 mutation in clinical samples

**Figure 1:**
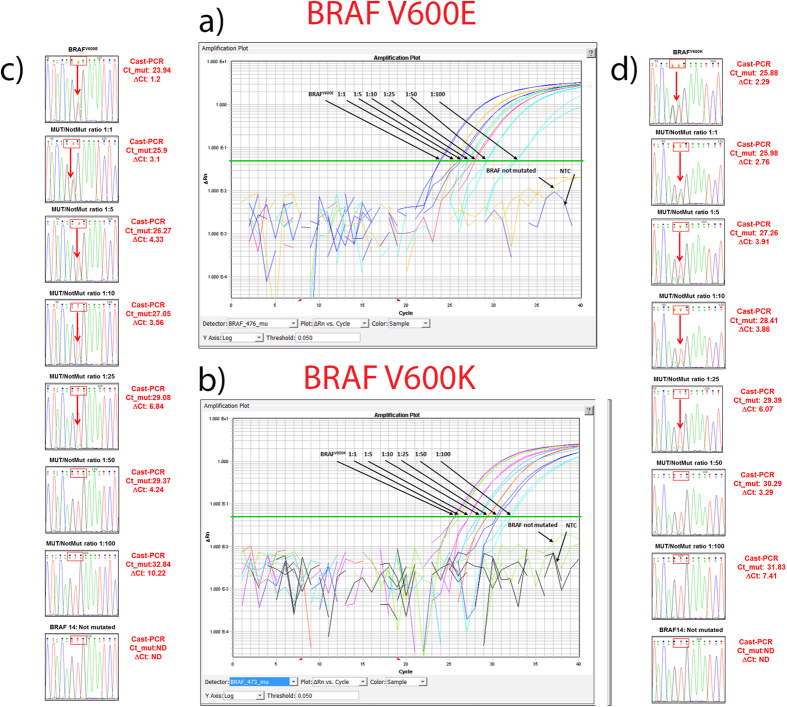
Evaluation of Cast-PCR and Sanger analysis Limit of Detection (LOD). For each of the *BRAF* mutations analyzed we determined the limit of detection by analyzing dilutions at different ratio of mutated (case 2 and case 23) and not mutated (case 40) clinical samples. **(a)** Cast-PCR plots for BRAF^V600E^ mutation. **(b)** Cast-PCR plots for BRAF^V600K^ mutation. **(c)** Sanger analysis of samples of different BRAF^V600E^:Not mutated ratio**; (d)** Sanger analysis of different BRAF^V600K^ : Not mutated ratios.

For each of the *BRAF* mutations analyzed we determined the Limit of Detection (LOD) of Cast-PCR and Sanger sequencing by constructing dilution curves which made use of both a single mutated (Mut) and not mutated (NMut) clinical samples. Each point was prepared by diluting the Mut sample with the NMut sample at different ratios (Mut/NMut range 1:1 to 1:100), in the attempt to set experimental conditions as close as possible to the routine diagnostic setting. The two clinical samples carrying a BRAF^V600E^ (case 2) and a BRAF^V600K^ (case 23) mutation respectively were chosen because they showed almost the same peak heights for both the mutated and not mutated alleles in two independent Sanger analyses, suggesting that they contain at least 50% of mutated alleles. Not mutated sample case 40 was used for both curves (Fig. 1a–d). The percentage of neoplastic cells was 90% for case 2, 50% for case 23 and 60% for case 40. Ct and ΔCt values for both curves are shown in Fig. 1a–d. In our setting, we found that the mutant alleles were well detectable up to the 1:100 Mut/NMut ratio for both case 2 (BRAF^V600E^), and case 23 (BRAF^V600K^) by Cast-PCR. Whereas, Sanger sequencing was not able to clearly detect the mutant alleles beyond the 1:25 Mut/NMut ratio.

### Molecular cloning of discrepant cases demonstrate high specificity of Cast-PCR in clinical samples

**Figure 2:**
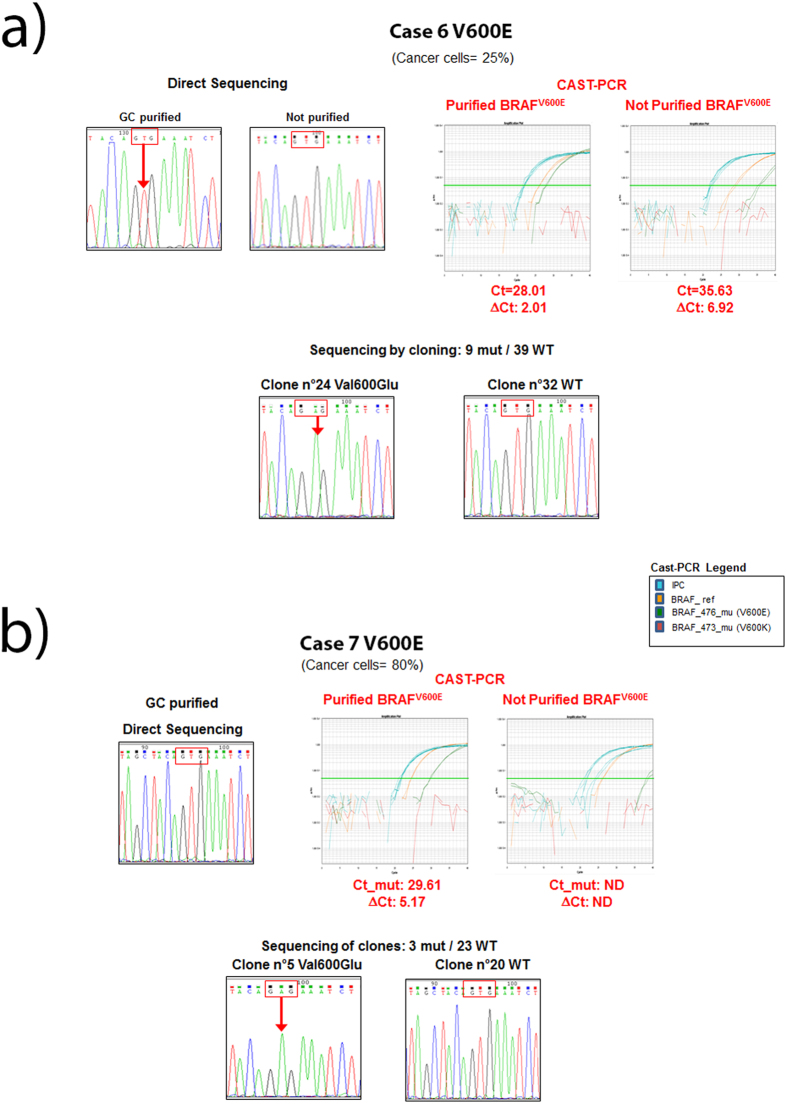
The effect of melanin content on *BRAF* mutation detection. (**a**) Case 6 did not show any mutation in the not purified sample by Sanger analysis as compared with the GC-purified correspondent sample. Cast-PCR plots show an amplification curve in both samples although Ct and ∆Ct values are higher in the not purified sample as compared with the GC purified. Cast-PCR results in the GC purified sample, were confirmed by molecular cloning. (**b**) Case 7 did not show any mutation even in the GC purified sample by Sanger analysis; on the other hand, Cast-PCR amplification was obtained exclusively upon GC-purification, and this result was further confirmed by molecular cloning.

To evaluate Cast-PCR performance we selected two of the discrepant cases, case 6 (cancer cells 25%) and case 7 (cancer cells 80%) with a sufficient DNA amount for sequencing by cloning analysis. The amplicon including codon 600 of the *BRAF* gene was cloned in the StrataClone plasmid Vector, and 39 clones for case 6 and 23 clones for case 7 were sequenced. For case 6, 9 out of 39 clones (23%) showed the BRAF^V600E^ mutation. (Fig. 2a). Whereas for case 7, 3 out of the 23 clones (13%) carried the BRAF^V600E^ variant (Fig. 2b). As control, we evaluated the not mutated case 42 by both Cast-PCR and Sanger analysis without finding mutation in the 40 clones analysed (data not shown).

### Detection of *BRAF* codon 600 mutations and melanin content

We evaluated the performance of both Sanger analysis and Cast-PCR before and after purification with GENECLEAN® II Kit in 29 samples from 22 cases (Table 4). GENECLEAN (GC) purification increased the percentage of cases successfully analysed from 27.3% (n = 6) to 95.4% (n = 21) for Sanger sequencing, and from 95.4% (n = 21) to 100% (n = 22) for Cast-PCR. Interestingly, melanin content may specifically affect the ability to identify *BRAF* codon 600 mutations, as shown in Table 4. Indeed, sample case 6 did not show any mutation in the not purified sample when analyzed by Sanger Sequencing, and was still uncertain after purification. Conversely, Cast-PCR was able to clearly demonstrate the presence of a BRAF^V600E^ mutation in both not purified and purified samples in most of the cases (Fig. 2a). The same mutation was further confirmed by molecular cloning. However, even Cast-PCR efficiency seems to be affected by melanin content. Indeed, ΔCt values were higher in not purified samples (Mean ± SD 2.55 ± 1.61) than in purified ones (Mean ± SD 3.60 ± 2.19), although this difference was not statistically significant (*p* value = 0.14). Moreover, case 7 showed a BRAF^V600E^ mutation by Cast-PCR only upon GC-purification.

### *BRAF* codon 600 mutations detection and tumour heterogeneity

**Figure 3:**
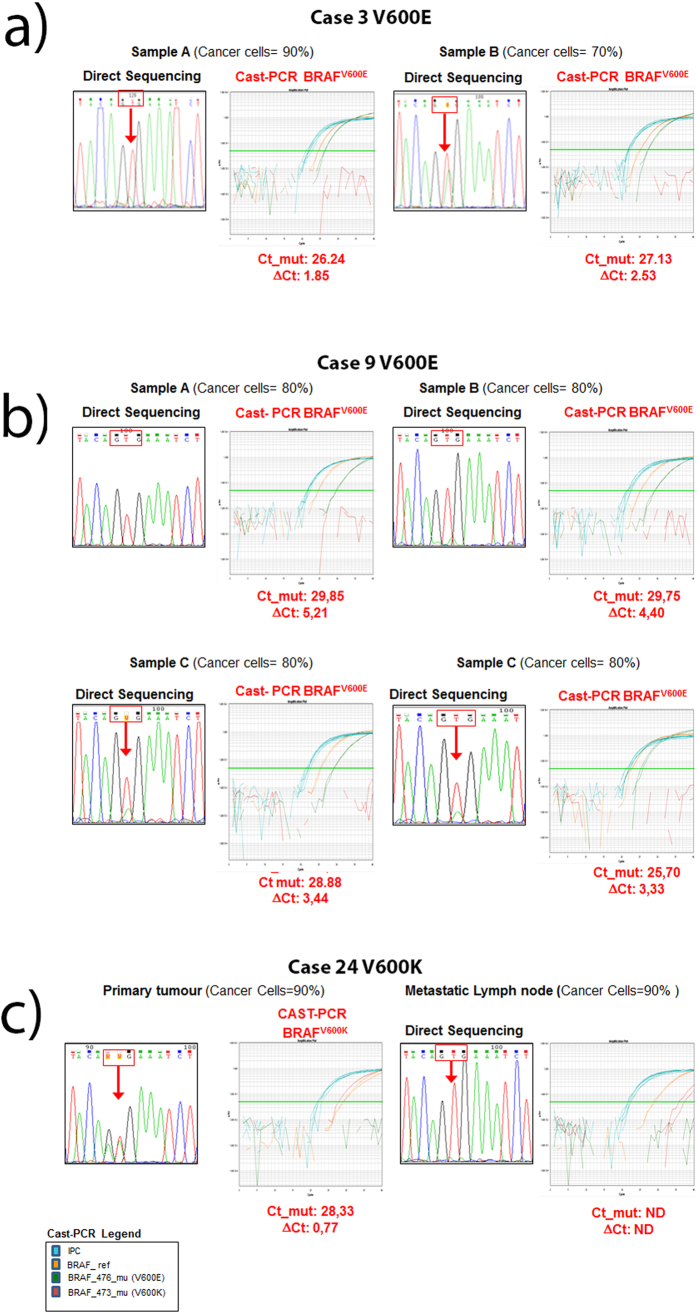
Intratumour and intertumor heterogeneity. (**a**) For case 3 two different regions from the same section were analysed. Both regions showed a BRAF^V600E^ mutation by Sanger sequencing as well as by Cast-PCR. However peak height in Sanger analysis was lower in sample A as compared to sample B, whereas Ct and ΔCt values were similar in both samples; (**b**) For case 24 the primary tumour and a synchronous metastatic lymphnode were analyzed. A BRAF^V600K^ mutation was detected in the primary tumour but not in the metastatic lymphnode by both Sanger analysis and Cast-PCR, (**c**) For case 9 four different regions from the same section were analysed. Sanger sequencing detected the BRAF^V600E^ mutation only in samples A and B, whereas Cast-PCR was able to detect the mutation in all four samples.

With respect to intratumour heterogeneity Cast-PCR showed concordant results in all samples for which different regions on the same section were available (n = 6) (Fig. 3a–c). Whereas, in case 9 Sanger sequencing was unable to detect the mutation in 1 out the four regions randomly selected on the section (Table 4) (Fig. 3b).

For 4 melanoma cases, different tissue blocks were analysed. Concordant results were demonstrated in 3 out of the four cases. Whereas, in case 24 a BRAF^V600K^ mutation was detected by Cast-PCR in the primary tumour but not in the metastatic lymph node (Table 4) (Fig. 3c).

### *BRAF* codon 600 mutational spectrum and correlation with clinicopathological characteristics

Of the 29 codon 600 mutations carriers identified by Cast-PCR, 21 (73%) were BRAF^V600E^ and 8 (27%) BRAF^V600K^. The Mean age of the subjects carrying a BRAF^V600E^ mutation was significantly younger (Mean ± SD 54.9 ± 14.1) as compared with patients harbouring a BRAF^V600K^ mutation (Mean ± SD 6.99 ± 13.6) (*p* = 0.023, t-test). Table 5 shows the distribution of mutation according to primary site. As expected, mutations were more common in cutaneous melanomas (n = 42) than other sites (n = 11). One mutation was identified in a gallbladder metastasis from a melanoma of unknown origin and the other in a melanoma of the iris.

### *BRAF* codon 600 mutations and treatment with Tyrosine Kinase Inhibitors (TKIs)

Follow up data were available for 19 of the 29 mutated cases. At this time, 12 of those patients are treated with TKIs, one patient refused treatment, and in two cases treatment was suspended for adverse effects. Of the treated patients, 2 were classified as responders, 4 partial responders, and 2 with stable disease after at least six months of TKIs administration. The remaining 4 patients showed stable disease after 3 months of treatment (Supplemental Table 1). It is of note that, at the time of our molecular investigation, sample from case 6 was a primary melanoma with a very low (25%) cancer cell content in which the presence of the BRAF^V600E^ mutation was identified only by Cast-PCR, whereas electropherograms could have led to a false negative result. This patients is currently under treatment with TKIs and is classified as a good responder. Moreover, for case 15 mutation could be detected only after GENECLEAN purification. This patient is currently under treatment with TKIs, and is showing stable disease. Of the 4 cases resulted as not mutated by Sanger analysis but mutated by Cast-PCR, we do not have clinical follow up data about one patient, and two did not start the treatment yet. In case 7, where the mutation was detected only by Cast-PCR and confirmed by molecular cloning, patient died four months after the molecular pathology diagnosis, but we do not know whether or not he received the treatment with TKIs. Patient 24, showing a mutation in the primary tumour but not in the metastatic lymphnode, is currently under treatment with TKIs although at a reduced dosage due to toxicity.

## Discussion

*BRAF* mutation analysis is now mandatory to identify patients affected by non resectable or metastastic melanoma who may benefit from Tyrosine Kinase Inhibitors. Herein, we report a methodological approach with the potentiality to fulfill the requirements which may impact on the efficiency of the *BRAF* mutation analysis in the routine clinical setting, including the standardization of the method itself, its sensitivity, specificity and capability to identify the mutations.

There is no generally accepted standard method for the determination of *BRAF* somatic mutations, and many methods are currently in use^29^. Sanger sequencing is considered by many as the gold standard for mutation detection because it is able to identify all sequence variants, but it has significant limitations due to the low sensitivity (20%), and often low quality results from FFPE specimens^30^,^31^. Thus, we support that the use of additional and more sensitive techniques may improve the ability to detect *BRAF* mutations in melanoma patients.

In this study we evaluated the putative use of Cast-PCR for the detection of BRAF^V600E^ and BRAF^V600K^ mutations in the clinical setting^25^,^26^,^27^,^28^,^29^,^30^,^31^,^32^. We found a 100% negative agreement between Cast-PCR and Sanger sequencing. Meanwhile, Cast-PCR identified 4 additional mutated cases, and clearly demonstrated the presence of mutation in two cases which resulted as uncertain by Sanger sequencing. Richter *et al.* also reported in an australian cohort of 93 melanoma cases that Cast-PCR is a high sensitive method for *BRAF* mutations detection as compared with Sanger sequencing, SSCA and HRM^27^. Moreover, in both our and Richter studies, the use of Cast-PCR seems to be crucial to minimize the risk of false negative results in tumour specimens with a low content of mutated DNA^27^. In our study however we have also supported these evidences by evaluating the limit of detection of Cast-PCR by using serial dilutions of real clinical samples for each of the *BRAF* mutations analyzed, instead of DNA obtained from cell lines or synthetic oligonucleotides. We believe that this approach would better reflect the limitation of *BRAF* testing in melanoma including the fact that we usually handle FFPE specimens with a variable percentage of cancer cells and often showing a marked intratumour heterogeneneity. As a result, by using the mixture of mutated (Mut) and not mutated (NMut) samples at different ratios in constructing our dilution curve, we identified the 1:100 Mut/NMut ratio as the limit of detection of both the BRAF^V600E^ and BRAF^V600K^ Cast-PCR probes. Since both mutated samples showed approximately the same peak heights in Sanger sequencing, we could assume that they contained approximately 50% of the mutated and 50% not mutated allele. This would set the sensitivity of Cast-PCR to 0.5% that it is close to the 0.1% sensitivity reported by the manufacturers and lower than 2% reported by Richter *et al.* as reported for the two samples showing discrepant results between sequencing and Cast-PCR^27^,^28^,^29^,^30^,^31^,^32^.

In our study we did not detect any rare *BRAF* mutation by sequencing analysis, thus the efficiency of Cast-PCR assays was only evaluated for the assays detecting BRAF^V600E^ and BRAF^V600K^ variants. Instead, Richter *et al.* identified 7 rare *BRAF* mutations by Sanger sequencing, SSCA and HRM that were not detected by Cast-PCR using the BRAF^V600E^ and BRAF^V600K^ assays. Of those mutations, four were BRAF^K601E^ (c.1801A > G, p-Lys601Glu), two were BRAF^V600E2^ (c.1799_1800delinsAA, p.Val600Glu) and one was a BRAF^V600R^(c.1798_1799GT > AG, p.Val600Arg)^27^. To date, the frequency of rare *BRAF* activating mutations reported in melanoma ranges from 5–12%. Moreover in preclinical studies and in a Phase I/II clinical trial the presence of those mutations has been associated with a variable degree of response to TKIs (either reduced cell proliferation or delay in progression). Hence, until further clinical trials will better clarify the role of rare *BRAF* mutations in mediating the response to TKIs, all possible mutations should be tested to avoid missing patients who may benefit from treatment with anti-BRAF TKIs^10^,^33^,^34^,^35^.

In this context, pyrosequencing is a more sensitive screening method as compared with Sanger sequencing; anyhow, it requires the use of proper and dedicated equipment, and specific assays still need to be designed for non-codon 600 mutations^19^,^20^,^21^. Moreover, there are currently no data about the performance of this technique in the clinical setting when the percentage of cancer cells drops close to its limit of detection. Thus, we can hypothesize that, while screening methods might be sufficient to detect all *BRAF* mutations in the presence of adequate percentage of tumour cells in a given sample, a combined approach with Cast-PCR would efficiently overcome the melanin content and tumour heterogeneity issues and guarantee a better genotypization of the patients.

Although the use of highly sensitive techniques improves our ability to detect minor mutated alleles in a given samples, it rises the question about the specificity of the technique. In our study we have assessed Cast-PCR specificity in two samples which resulted as harbouring BRAF^V600E^ mutations by Cast-PCR but uncertain (case 6) or negative (case 7) by Sanger sequencing. These samples were analysed by cloning and sequencing of the clones, and in both cases we were able to detect the same mutation identified by Cast PCR in the minority of the clones. As further control, case 42 was confirmed as no mutated by all the methods employed. Of course, the ability of Cast-PCR to detect very low level of *BRAF* mutations is significantly helpful in the evaluation of samples with low percentage of neoplastic cells. On the other hand, the clinical relevance of the detection of minor mutated alleles in samples with high percentage of neoplastic cells is currently unknown. In our study, all but one of the discrepant (n = 4) or uncertain (n = 2) cases had more than 70% of neoplastic cells. Case 6 classified as uncertain by Sanger sequencing contained 25% of neoplastic cells. The patient is currently under treatment with TKIs, taking significant benefits from it. Of the remaining 5 cases, two patients were not treated with TKIs and for another no follow up data are available. Patient 7 was affected by lung metastases and died four months after testing, but there are no data on whether or not he was treated with TKIs. It is known that the use of TKIs in patients without *BRAF* codon 600 mutation further activates the MAPK pathway resulting as detrimental for patients survival^36^. Thus, our results suggest that the issue of whether the presence of very low level of *BRAF* mutations in cases with high percentage of neoplastic cells correlates with response to TKIs should be addressed in multicentre post-marketing surveillance.

Although melanoma is mostly of cutaneous origin, it can also occur in various extracutaneous sites including ocular melanomas, mucosal and leptomeningeal melanomas^1^. The frequency of *BRAF* codon 600 mutations in non-cutaneous is significantly lower as compared with cutaneous melanomas^37^. In our series we found 2 mutations among the non cutaneous melanoma cases. In particular, we identified the BRAF^V600E^ mutation in a melanoma of the iris confirming a previous study which suggested that *BRAF* mutations do exist in this ocular melanoma subtype as compared with posterior uveal melanoma where *BRAF* mutations have never been reported^38^,^39^.

Among the technical issues related to *BRAF* mutation detection in melanoma, melanin content is overlooked. Indeed, melanin coisolates with DNA and it is a strong inhibitor of DNA polymerase^40^. Several studies in the forensic field evaluated the diagnostic performance of analytical methods in relation to the ability of extraction/purification techniques to reduce melanin content^41^,^42^. However, to the best of our knowledge, the effect of melanin content in the detection of *BRAF* mutation in melanoma sample has never been addressed before. To maximize DNA recovery even from small clinical samples, we routinely use a phenol chloroform protocol for DNA extraction, followed by glass milk^28^,^43^ purification as previously validated in uveal melanoma samples. Thus, on a selected number of cases (n = 22), we compared the performance of Cast-PCR and Sanger analyses before and after purification. As expected, melanin content strongly affected the number of cases successfully analysed by Sanger sequencing, and interpretable electropherograms were obtained only in 4% of not purified cases. Likely due to the use of less amount of starting DNA, Cast-PCR is less affected by melanin content, with only one of the not purified cases failing the analysis. However, melanin content was able to affect the sensitivity of both techniques. Before purification, 1 out of the 4 cases successfully analysed by Sanger analysis would have been genotypized as not mutated, as well as 1 out of the 21 cases analysed by Cast-PCR would have led to a false negative results. Thus, our results strongly suggest that diagnostic laboratories should evaluate whether their pre-analytical procedures are able to reduce the impact of melanin content on subsequent analytical methods to avoid the risk of false negative results.

Several studies have demonstrated the presence of BRAF^V600^ heterogeneity in melanoma^22^,^23^,^24^,^25^,^44^. We identified one case showing discrepant results in 2 out of the 4 region obtained separately from the same block (case 9) by Sanger analysis. However, in this case, Cast-PCR was able to detect the mutations in all specimens tested. As regard to intertumour variability 1 out of the 2 cases (case 24) for which paired primary tumor and synchronous lymph node metastases were available showed a BRAF^V600K^ mutation by both Sanger and Cast PCR analyses in the primary tumour but not in the metastases. Although we evaluated intertumoral and intratumoral heterogeneity on a limited number of cases (n = 6 and n = 4 respectively), our results suggest that the use of Cast-PCR may overcome some of the issues related to the presence of different subclones within the tumour. On the other hand, no conclusions can be made for issues related to differences among primary and metastatic tumours, and whether they may represent an intrinsic mechanism of resistance to *BRAF* codon 600 TKIs.

Overall our results demonstrated that Cast-PCR is a sensitive and specific method for the detection of BRAF^V600E^ and BRAF^V600K^ mutations in melanomas. In addition, Cast-PCR is less affected by melanin content and by intratumour heterogeneity, thus representing a robust and reliable method for the selection of melanoma patients candidate to treatment with TKIs.

## Materials and Methods

### Patients and samples

We analysed by Cast-PCR and Sanger sequencing analysis 54 specimens (53 melanoma and 1 Spitz nevus) as part of the routine clinical screening for the identification of candidates to TKIs treatment. Among them, 27 were male (50%) and 27 females (50%), and the mean age at diagnosis was 59 years (range 34–82 years). Forty-two cases were cutaneous melanoma (78%), 4 mucosal (7%), 3 uveal (6%), 1 iris melanoma (1%), and 3 metastatic melanoma from an unknown site of origin (6%). Prior written and informed consent was obtained from each patient in accordance with the guidelines approved by the IRCCS “Casa Sollievo della Sofferenza” Ethical Committee. *BRAF* molecular testing was performed in agreement with the Italian guidelines as approved by the AIOM (Italian Association of Medical Oncology)/SIAPEC (Italian Society of Pathology and Cytopathology) Working Group (https://testbiomolecolari.it/system/files/allegati/Raccomandazioni_BRAF.pdf)

For each sample, tumour cell content was assessed by a referral pathologist (P.G.) on a histological section stained with hematoxylin and eosin. For tumor samples with a percentage of tumor cells < 70%, a macrodissection of the specimen was performed where possible, in order to increase the relative content of tumour cells. The percentage of cancer cells after macrodissection ranged from 10% to 90%. In 5 cases different regions of the same section were macrodissected and in 4 cases samples from different tissue blocks were evaluated (Supplemental Table 1). Overall, 66 Formalin-fixed Paraffin-embadded (FFPE) specimens from the 54 patients were analysed.

### DNA extraction and purification

DNA was extracted by phenol chloroform as described previously^43^. Briefly, tumour tissue was digested with sodium dodecyl sulfate/proteinase K, extracted with phenol/chloroform, and purified by GENECLEAN® II kit (Vista)according to the manufacturer’s instructions^28^,^43^.

### Competitive allele specific TaqMan-PCR (Cast-PCR)

Samples were analysed by Cast-PCR using the BRAF_476_mu and BRAF_473_mu probes for the detection of V600E (c.1799T > A, p.Val600Glu), and V600K mutations (c.1798_1799delinsAA, p. Val600Lys), respectively (Life Technologies). Master mixes were prepared as recommended by the manufacturer and distributed in a 384-well plate. 10 ng of DNA samples were added to each well and the reaction was carried out in an ABI PRISM 7900HT Sequence Detection System (Life Technologies). The PCR conditions comprised an initial denaturation step of 10 minutes at 95 °C, followed by 5 cycles of 15 sec denaturation at 95 °C, and one minute extension at 58 °C. This was followed by 40 cycles of 15 sec denaturation at 95 °C and one minute extension at 60 °C. Real time data were collected during the last 40 cycles of amplification and analysed using the Mutation DetectorTM software v.2.0 (Life Technologies).

### Fluorescence based direct sequencing analysis (Sanger sequencing)

Primer pairs were designed to amplify a fragment spanning the catalytic domain of *BRAF* including codon 600. Primers sequence was as follows: forward primer, 5′- CATAATGCTTGCTCTGATAG -3′ and reverse primer, 5′- GTAACTCAGCAGCATCTCAG -3′. Sequence reactions were performed on an automated sequencer (ABI 3100; Life Technologies) using the ABI-PRISM Big-Dye Terminator Cycle Sequencing Ready Reaction kit (Life Technologies) and analyzed by BLAST and manual review of chromatograms.

### Dilution Curves construction

The limit of detection (LOD) of Cast-PCR and Sanger sequencing was evaluated by mixing codon 15 mutated (mut) DNA samples from two patients with a DNA sample from not mutated patients at different ratios. Mutated cases 2 and case 23 were chosen because the mutated and not mutated alleles showed almost the same peak heights in two independent Sanger sequencing analyses. Case 40 was chosen among the not mutated samples by both Sanger sequencing and Cast-PCR because of the amount of DNA available to dilute samples for both curves. Starting from 100 ng of DNA, mutated samples were diluted with the not mutated sample starting from 100 ng the following ratio 1:1, 1:5, 1:10, 1:25, 1:50, 1:100.

### Cloning-sequencing analysis

For the cloning and sequencing analysis, the genomic region of the chromosome 7: 140453013-140453255 *BRAF* gene, including codon 600 was amplified with the same primer pair used for direct sequencing analysis. The PCR products were then ligated in StrataClone Vector Mix amp/kan (Agilent Technologies). The recombinant plasmids were used to transform StrataClone SoloPack Competent Cells according to the manufacturer’s instructions. Twenty selected clones were used directly as template for the PCR with the T3/T7 primer pair and subjected to Sanger sequencing.

### Statistical analyses

Agreement between Cast-PCR with Sanger sequencing analysis was measured using κ-statistics. Wilson’s ellipse method was used to determine positive and negative agreement 95% confidence intervals. The Student t-test was used^43^,^44^ to compare continuous variables.

## Additional Information

**How to cite this article**: Barbano, R. *et al.* Competitive allele-specific TaqMan PCR (Cast-PCR) is a sensitive, specific and fast method for BRAF V600 mutation detection in Melanoma patients. *Sci. Rep.* **5**, 18592; doi: 10.1038/srep18592 (2015).

## Supplementary Material

Supplementary Information

## Figures and Tables

**Table 1:** Comparison between Cast-PCR and Sanger sequencing mutation detection rates in the 54 patient cases.

Results	Sanger		Cast-PCR	
	n	%	n	%
Mutated	**22**	**40.7**	**29**	**53.7**
Not mutated	29	53.7	25	46.3
Uncertain	2	3.7	0	0
Not determined	1	1.9	0	0

**Table 2:** Discrepant cases between Cast-PCR and Sanger analysis.

Case	SANGER	Cast-PCR	ΔCt	% Cancer cells	Sex	Tumour type	Age	Status	TKIs
1	not mutated	V600E	2.20	90%	F	CM	69	unknown	unknown
5	not mutated	V600E	0.82	50%	F	CM	61	alive	NO
6	uncertain	V600E	2.01	25%	F	CM	75	alive	YES
7	not mutated	V600E	5.59	80%	M	CM	53	dead	unknown
11 primary	not mutated	V600E	4.69	90%	F	CM	81	unknown	unknown
11 lymphnode	not mutated	V600E	2.72	95%					
22	uncertain	V600K	6.93	70%	M	CM	80	alive	NO

**Table 3:** Evaluation of negative and positive agreement among Cast-PCR and Sanger analyses.

		Cast-PCR	
		Not mutated	Mutated
Sanger	Not mutated	n = 25	n = 4
	Mutated	n = 0	n = 24

**Table 4:** Effect of GeneClean purification and tumour heterogeneity on Cast-PCR and Sanger sequencing sensitivity.

Case	SANGER		Cast-PCR			
	GC-purified	Not purified	GC-purified	ΔCT	Not purified	ΔCT
2	V600E	Failed	V600E	1.95	V600E	1.9
3 sample A	V600E	Failed	V600E	1.85	V600E	2.53
3 sample B	V600E	Failed	V600E	2.22	V600E	2.32
4	V600E	Failed	V600E	1.56	V600E	1.46
6	uncertain	Not mutated	V600E	2.01	V600E	6.92
7	Not mutated	Failed	V600E	5.17	Not mutated	
8 sample A	V600E	V600E	V600E	1.74	V600E	2.09
8 sample B	V600E	V600E	V600E	2.06	V600E	1.56
9 sample A	Not mutated	Failed	V600E	5.21	Failed	
9 sample B	Uncertain	Failed	V600E	4.40	Failed	
9 sample C	V600E	Failed	V600E	3.44	Failed	
9 sample D	V600E	Failed	V600E	3.33	Failed	
10 sample A	V600E	V600E	V600E	1.58	V600E	2.30
10 sample B	V600E	V600E	V600E	0.10	V600E	1.32
11 primary	Not mutated	Failed	V600E	4.69	V600E	5.00
11 lymphnode	Not mutated	Failed	V600E	2.72	Not done	
15	V600E	Failed	V600E	3.33	V600E	5.03
22	uncertain	Failed	V600K	6.93	V600K	8.3
23	V600K	V600K	V600K	2.4	V600K	3.22
24 primary	V600K	Not done	V600K	0.77	Not done	
24 lymphnode	Not mutated	Not done	Not mutated		Not done	
25	Failed	Failed	V600K	2.04	V600K	6.03
29	V600K	V600K	V600K	3.77	V600K	4.05
31 sample A	Not mutated	Failed	Not mutated		Not mutated	
31 sample B	Not mutated	Not done	Not mutated		Not done	
32	Not mutated	Failed	Not mutated		Not mutated	
38	Not mutated	Failed	Not mutated		Not mutated	
39	Not mutated	Failed	Not mutated		Not mutated	
40	Not mutated	Failed	Not mutated		Not mutated	
41 sample A	Not mutated	Failed	Not mutated		Not mutated	
41 sample B	Not mutated	Not done	Not mutated		Not done	
41 sample C	Not mutated	Not done	Not mutated		Not done	
42	Not mutated	Not mutated	Not mutated		Not mutated	
43 block 1	Not mutated	Failed	Not mutated		Not mutated	
43 block 2	Not mutated	Not done	Not mutated		Not done	

**Table 5:** Mutational spectrum of *BRAF* codon Val600 mutations according to melanoma subtype.

		Mutated		Not Mutated	
Melanoma Subtype		n	%	n	%
Cutaneous	(n = 42)	27	64.3	15	35.7
Mucosal	(n = 4)	0	0	4	100
Eye	(n = 4)	1	25	3	75
Occult*	(n = 3)	1	33.3	2	66.7

^*^Visceral metastases from melanoma of unknown origin.
