# Supplementary material for: Competitive allele-specific TaqMan PCR (Cast-PCR) is a sensitive, specific and fast method for BRAF V600 mutation detection in Melanoma patients
Source: Sci Rep. 2015 Dec 22;5:18592. doi: 10.1038/srep18592 (PMC4686972; doi:10.1038/srep18592)
Supplement: Supplementary Information [file srep18592-s1.pdf]

Competitive allele-specific TaqMan PCR (Cast-PCR) is a sensitive, specific and fast method for BRAF V600 mutation detection in Melanoma patients.

Raffaela Barbano[1], Barbara Pasculli[1], Michelina Coco[1], Andrea Fontana[2], Massimiliano Copetti[2], Michelina Rendina[1,3], Vanna Maria Valori[3], Paolo Graziano[4], Evaristo Maiello[3], Vito Michele Fazio[1,5], Paola Parrella[1*].

[1]Laboratory of Oncology, [2]Biostatistic Unit, [3]Department of Oncology, [4]Department of Pathology, IRCCS Casa Sollievo della Sofferenza, San Giovanni Rotondo (FG) 71013; [5]CIR Laboratory for Molecular Medicine and Biotechnology, University Campus Biomedico, Rome 00128, Italy.

Supplemental Table 1.

Supplemental Table 1. Clinicopathological characteristics and mutational analysis results for the 54 patients analysed by direct sequencing and Cast-PCR

CASE	Gender	Age	Tumor Type	primary site	Regional Lymphnode	Progression	site progression	sample analyzed	% tumor cell	SANGER	Cast-PCR	Cast-PCR ΔCt	anti-BRAF treatment	Response to anti-BRAF treatment	TREATMENT NOTES
1	F	59	Cutaneus Melanoma	leg	negative	YES	Skin	primary site	80%	Not Mutated	V600E	2.1	NO		
2	F	41	Cutaneus Melanoma	leg	positive	YES	brain lung	lymph node	90%	V600E	V600E	1,95	not available		
3	M	43	Cutaneus Melanoma	trunk	negative	yes	Skin, Lymph node	primary site	A= 90% B= 70%	V600E	V600E	A= 1.85 B= 2.22	YES	Partial Response	Progression after 7 months
4	F	57	Cutaneus Melanoma	trunk	sentinel lymph node	YES	Lymph node	primary site	75%	V600E	V600E	1.56	not yet		
5	F	61	Cutaneus Melanoma	trunk	positive	YES	Lymph node	primary site	50%	Not Mutated	V600E	0.82	NO		
6	F	75	Cutaneus Melanoma	trunk	positive	YES	Skin, Lymph node	primary site	25%	low height peak	V600E	2.01	YES	Response	on going
7	M	53	Cutaneus Melanoma	trunk	positive	YES	Lung	lymph node	80%	Not Mutated	V600E	5.17	not available		
8	M	41	Cutaneus Melanoma	leg	positive	YES	Skin	primary site	A= 60%; B= 50%	V600E	V600E	A= 1.74: B= 2.06	not available		
9	M	33	Cutaneus Melanoma	trunk	positive	YES	Liver	primary site	A, B, C and D 80%	V600E	V600E	A=5.21; B=4.40; C=3.44; D=3.33	YES	Stable Disease	at 3 months
10	F	53	Cutaneus Melanoma	cheek	positive	yes	Lymph node	lymph node	A and B 90%	V600E	V600E	A= 1.58; B=0.10	YES	Toxicity	Suspended for toxicity after 1 month
11	F	81	Cutaneus Melanoma	trunk	negative	YES	primary site; lymphnode	Primary (T1); lymphnode (T2)	T1=90%; T2=95%	Not Mutated	V600E	T1=4.69; T2=2.72	NO		
12	F	48	Cutaneus Melanoma	trunk	positive	YES	Breast	breast	80%	V600E	V600E	A=1.72; B=2.04	not available		
13	M	72	Cutaneus Melanoma	trunk	negative	YES	Skin	skin	80%	V600E	V600E	1.71	YES	Partial Response	Progression after 6 months
14	F	42	Cutaneus Melanoma	trunk	positiveitive	YES	Lymph node	lymph node	50%	V600E	V600E	2.01	YES	Response	at 3 months
15	F	62	Cutaneus Melanoma	trunk	positive	YES	Breast	breast	90%	V600E	V600E	0.21	YES	Stable Disease	at 3 months
16	M	62	Cutaneus Melanoma	trunk	positive	YES	Lymph node	lymph node	90%	V600E	V600E	2.26	NO		
17	F	61	Cutaneus Melanoma	trunk	positive	YES	Brain, Lung	lymph node	90%	V600E	V600E	2.14	YES	Toxicity	Suspended for toxicity after 3 months
18	M	51	Cutaneus Melanoma	unknown	positive	YES	Lymph node	lymph node	60%	V600E	V600E	3.33	NO		
19	M	44	Cutaneus Melanoma	not available	positive	YES	Lymph node	lymph node	60%	V600E	V600E	1.88	not available		
20	M	35	Cutaneus Melanoma	Neck	not available	not available	not available	primary site	90%	V600E	V600E	1.73	YES	Response	at 3 months
21	F	74	Cutaneus Melanoma	LEG	positive	YES	Skin	skin	80%	V600K	V600K	3,17	not available		
22	M	80	Cutaneus Melanoma	arm	positive	YES	Skin	skin	70%	low height peak	V600K	6,13	NO		
23	M	52	Occult Melanoma	gallbladder	positive	not available	not available	gallbladder	50%	V600K	V600K	2.4	not available		
24	M	46	Cutaneus Melanoma	trunk	sentinel lymph node	YES	primary site; lymphnode	primary (T1); lymph node (T2)	T1=90%; T2=90%	T1 V600K; T2 not mut	T1 V600K; T2 not mutated	T1= 0.77	YES	Toxicity, reduced dosage	on going
25	M	77	Uveal Melanoma	Iris	not available	YES	Liver	primary site	80%	Failed analysis	V600K	2.04	NO		Refused treatment
26	F	84	Cutaneus Melanoma	scalp	not available	NO	NO	primary site	80%	V600K	V600K	2.06	not yet		
27	M	81	Cutaneus Melanoma	arm	positive	YES	Skin	skin	60%	V600K	V600K	2.52	not available		
28	F	66	Cutaneus Melanoma	cheek	not available	not available	not available	syncronous lung metastases	80%	V600K	V600K	1.12	YES	Stable Disease	at 3 months
29	M	71	Cutaneus Melanoma	trunk	negative	yes	Lymph node	lymph node	90%	V600K	V600K	3.77	YES	Stable Disease	at 3 months
30	M	62	Occult Melanoma	unknown	positive	YES	Retroperitoneal mass	Retroperitoneal mass	75%	Not Mutated	Not Mutated				
31	M	63	Cutaneus Melanoma	ear	positive	YES	Liver	primary site	A=50%; B=70%	Not Mutated	Not Mutated				
32	M	21	Spitz Nevous	leg	negative	NO	NO	primary site	50%	Not Mutated	Not Mutated				
33	F	35	Cutaneus Melanoma	trunk	negative	NO	NO	primary site	30%	Not Mutated	Not Mutated				
34	M	68	Cutaneus Melanoma	leg	negative	YES	Skin	skin	60%	Not Mutated	Not Mutated				
35	F	60	Mucosal Melanoma	nose	not available	YES	Bone	primary site	10%	Not Mutated	Not Mutated				
36	M	82	Occult Melanoma	lymph nodes	positive	YES	Syncronous bone metastases	lymph node	80%	Not Mutated	Not Mutated				
37	M	53	Mucosal Melanoma	rectum	positive	YES	not available	primary site	40%	Not Mutated	Not Mutated				
38	F	44	Uveal Melanoma	choroid	not available	YES	Liver	primary site	90%	Not Mutated	Not Mutated				
39	F	74	Cutaneus Melanoma	cheek	positive	YES	Brain	primary site	95%	Not Mutated	Not Mutated				
40	F	74	Mucosal Melanoma	nose	not available	YES	Pleura	pleura	60%	Not Mutated	Not Mutated				
41	M	69	Cutaneus Melanoma	arm	not available	YES	Skin	primary site	A, B and C 90%	Not Mutated	Not Mutated				
42	F	38	Cutaneus Melanoma	trunk	positive	YES	Peritoneum	primary site	90%	Not Mutated	Not Mutated				
43	F	79	Cutaneus Melanoma	acral	negative	YES	Skin	skin	80%	Not Mutated	Not Mutated				
44	F	34	Cutaneus Melanoma	leg	negative	NO	NO	primary site	40%	Not Mutated	Not Mutated				
45	M	28	Uveal Melanoma	choroid	not available	YES	Liver	primary site	90%	Not Mutated	Not Mutated				
46	F	58	Mucosal Melanoma	vulva	not available	not available	NO	primary site	90%	Not Mutated	Not Mutated				
47	M	54	Cutaneus Melanoma	arm	positive	YES	spleen	spleen	75%	Not Mutated	Not Mutated				
48	F	60	Cutaneus Melanoma	acral	negative sentinel	YES	skin	primary site	80%	Not Mutated	Not Mutated				
49	M	55	Uveal Melanoma	choroid	not available	YES	LIVER	metastases	80%	Not Mutated	Not Mutated				
50	F	85	Cutaneus Melanoma	not available	positive	YES	lung	lung	75%	Not Mutated	Not Mutated				
51	M	72	Cutaneus Melanoma	leg	positive	YES	Brain, Skin	primary site	80%	Not Mutated	Not Mutated				
52	F	81	Cutaneus Melanoma	nose	not available	not available	not available	primary site	70%	Not Mutated	Not Mutated				
53	F	74	Cutaneus Melanoma	cheek	not available	NO	NO	primary site	70%	Not Mutated	Not Mutated				
54	M	54	Cutaneus Melanoma	trunk	positive	NO	NO	lymph node	30%	Not Mutated	Not Mutated				
